# The Effect of Superficial Oregano Essential Oil Application on the Quality of Modified Atmosphere-Packed Pork Loin

**DOI:** 10.3390/foods12102013

**Published:** 2023-05-16

**Authors:** Weronika Zduńczyk, Katarzyna Tkacz, Monika Modzelewska-Kapituła

**Affiliations:** Department of Meat Technology and Chemistry, Faculty of Food Sciences, University of Warmia and Mazury in Olsztyn, Plac Cieszyński 1, 10-719 Olsztyn, Poland; weronika.zdunczyk@uwm.edu.pl (W.Z.); ktkacz@uwm.edu.pl (K.T.)

**Keywords:** colour, meat, food, packaging, shelf-life, quality

## Abstract

During meat storage, changes in the meat colour occur, making it less intensive and red. The present study was aimed at investigating the effect of oregano EO applied directly on the surface of fresh pork on its quality, with a special emphasis on the colour. In the study, an oregano essential oil in concentrations of 0.5% and 1.0% (*v*/*v*) was used on the surface of pork loins (1.5% *v*/*w*) packed in a modified atmosphere during 15-d storage at 4 °C. The application of oregano EO in the concentration of 1.0% increased lightness and hue and decreased redness compared to the control, whereas the concentration of 0.5% did not affect the pork colour. EO did not affect pH, free water content, purge and cooking losses, cooked meat juiciness and tenderness; however, it gave the meat a distinctive herbal aroma and taste. The antimicrobial effect of 1% EO was noted only on the 15th day. Therefore, the application of oregano essential oil is not recommended to protect the colour of raw pork nor to prolong its shelf-life; however, it might be used to obtain a new product with a specific herbal aroma and taste, with modifications in water-holding capacity of the meat.

## 1. Introduction

Pork meat is a food product popular worldwide, which results from culinary tradition as well as its nutritional value. In this respect, pork is considered a source of protein, minerals (iron, zinc, selenium, magnesium, phosphorus, potassium) and vitamins (thiamine, riboflavin, niacin, pantothenic acid, choline, B_6_ and B_12_) [1]. Meat having such a complex and nutritious composition is susceptible to detrimental changes during storage. Apart from changes of a microbial origin, oxidation of proteins, lipids and myoglobin occur on the meat surface, which results in modification of the meat quality, including its colour [2,3]. The colour of fresh meat is one of the most important quality attributes. Based on fresh meat colour, consumers make their decision about purchases. Therefore, a desirable vivid, light-pink colour of pork loin during the whole storage period until the meat is sold is so important. 

In order to extend the shelf-life of meat, including pork, appropriate methods of packaging are needed [4]. In the meat industry, commonly used packaging methods are packaging in a modified atmosphere (MAP) and in a vacuum. Comparing these methods, MAP is more effective in controlling the growth of *Pseudomonas* spp., psychrotrophic aerobic bacteria, lactic acid bacteria, *Enterobacteriaceae*, moulds and yeast [5,6]. However, MAP-packed meat is characterized by a higher lipid oxidation rate and colour deterioration than vacuum-packed [5]. Nevertheless, MAP still is commonly used by pork producers to pack fresh pork, and therefore new methods for preserving the quality of meat packed in this system are needed. One of the methods might be the application of essential oils (EO), which have antimicrobial and antioxidant properties [7,8,9]; moreover, they are safe, natural and environmentally friendly [10]. Therefore, nowadays, there is more and more research that focuses on the application of EO to prolong the shelf-life of meat products while preserving their appropriate appearance and sensory quality [11,12].

The effect of EO on the quality of meat depends not only on its origin (plant) and composition but also on its concentration. Moreover, the product’s characteristics (such as microbiota—its counts and composition, water activity, pH), as well as storage conditions (temperature, atmosphere composition) [13,14], affect EO effectiveness, and all of them should be taken into consideration when an EO is applied in a particular product. Boskovic et al. [15], who studied the effect of thyme EO (from 0.3% to 0.9%) on minced pork packed in MAP and vacuum, showed that the best antibacterial effect was achieved by a combination of MAP and 0.9% thyme EO, whereas the most acceptable were samples that contained 0.3% of the EO. In turn, van Haute et al. [6] compared the effect of cinnamon EO in a combination of packaging methods (vacuum and MAP) and noted that it was different for fresh pork and fresh salmon (it was more effective in pork samples). Therefore, results obtained in experiments conducted on different meat types or under different storage conditions might not be easily transferred to practice for different meat, EO and storage conditions. This indicates the need for further studies, which will broaden the knowledge about the possibility of using EO in the meat industry. 

In the present study, oregano essential oil was used based on preliminary studies in which different essential oils (clove, marjoram, coriander, juniper, oregano, rosemary) were tested in pork in different proportions. The oregano oil application in concentrations of 0.5% and 1.0% produced pork with the most desirable sensory quality; therefore, these concentrations were used in this study. Generally, oregano EO is extracted from oregano (*Origanum vulgare*) and shows strong bio-activeness, including antimicrobial, anti-inflammatory and antioxidant properties due to the presence of carvacrol and thymol. When used in meat products, it shows the growth inhibition of *Listeria monocytogenes*, *Staphylococcus aureus*, *Pseudomonas aeruginosa* and *Alicyclobacillus* [3,16,17,18]. In the present study, oregano EO was applied to pork obtained from animals, which were raised without antibiotics (RWA). This meat is regarded as a safer, premium option offered to consumers; therefore, it is vitally important to preserve its quality, with a special focus—its colour. According to the information obtained from the producer of the pork, during the storage in MAP (which is used to pack the RWA meat in the plant), the colour of the pork becomes less vivid, which might adversely affect consumers’ willingness to buy the meat. The shelf-life of the MAP-packed pork used in the study (as declared by the producer) is 14 days in refrigeration conditions of 4 °C. The meat industry puts a lot of effort to increase the shelf-life of fresh meat. Therefore, in the present study, analyses were carried out also the next day (15th) after the expiration date to determine if the extension of the date is possible by using EO. Indeed, studies in which EO are used very often focus on determining the optimal EO concentration that is needed for extending the shelf-life without an adverse effect on sensory quality [7,12,14,16,19]. However, Osimani et al. [7] showed that the EO concentrations that are able to inhibit bacterial growth might mask the symptoms of quality deterioration and make them not detectable to consumers. This might lead to the consumption of a low-quality product, which might not be safe. In the present study, apart from colour changes, changes in pH, water holding capacity (free water content and losses), as well as total viable counts and sensory quality of cooked meat were determined to provide a complex quality characteristic of the product and its safety. 

Therefore, the aim of this study was to determine the effect of oregano EO applied directly on the surface of fresh pork on its quality, with a special emphasis on the colour. The hypothesis that the application of oregano essential oil on the surface of fresh pork loin will preserve its colour for up to 15 days of storage was tested. 

## 2. Materials and Methods

### 2.1. Pork and Sample Preparation

Material for the research was kindly obtained from a meat supplier (Goodvalley Polska, Przechlewo, Poland). Pork loin (*longissimus lumborum* muscles) originated from nine DanBred porkers which were a crossbred of ♂ Duroc x ♀ (♂ Landrace x ♀ Yorkshire), which were raised without the use of antibiotics during fattening (RWA). The animals were slaughtered at the age of 24 weeks, with an average weight of 115 kg. The muscles used in the study (n = 9) were obtained from three different batches, 24 h after slaughter. Each muscle (average weight 1191.86 g ± 4.19) was sliced into 14 slices 1.5 cm thick. The slices were randomly distributed into three groups: control (without the application of oregano essential oil), EO 0.5 (samples in which oregano oil at 0.5% concentration was used) and EO 1.0 (samples in which oregano oil at 1.0% concentration was used).

In the study, a commercially available ORE2E00 (Essence Sp. z.o.o., Warsaw, Poland) oregano essential oil (EO) was used. According to the producer’s information, the oil was obtained as a result of the steam distillation of dried *Origanum vulgare* L. leaves. The oil contained carvacrol: 75%, gamma-terpinen: 5%, para-cymen: 4%, linalool: 3% [https://essence-eu.pl/olejki-eteryczne.html (accessed on 20 June 2021)]. The oregano essential oil in concentrations of 0.5% and 1.0% (*v*/*v*) was used. These concentrations were used based on our previous studies, in which higher concentrations produced pork with too intensive of a herbal aroma. The solutions were prepared by mixing 0.5 mL of the oregano EO with 99.5 mL of distilled water to obtain a concentration of 0.5% and 1.0 mL of the EO with 99.0 mL of distilled water to obtain a concentration of 1.0%. Although essential oils are not soluble in water, we used it as a medium, because an ethanol solution produced a meat surface discolouration as noted in our preliminary studies. Obtained solutions were spread over the surface of each meat piece using disposable atomizers in the amount of 1.5% with respect to the weight of each pork loin slice. Each pork loin slice was weighed to calculate the amount of the solution needed to be spread. The aqueous solution of the oregano EO was shaken well before its application on the pork loin slice surface. Using the above-described solutions and amount spread on the surface, the actual concentration of the oregano essential oil on the pork loin surface was 0.0075% and 0.015% when using 0.5% and 1.0% of the oregano EO solutions, respectively.

After, EO application samples were packed individually on plastic trays and packed in a modified atmosphere (70% O_2_/25% CO_2_/5% N_2_) using an automatic packaging machine Traysealer A6 SEALPAC (Sealpac, Oldenburg, Germany). MAP composition was monitored after packaging and during storage by Oxibaby 6.0 (Witt-Gasetechnik GmbH & Co KG, Salinger Feld, Germany). The packed samples were stored in a refrigerator (Asber Ecp-G-1402 Glass, Asber, Palmiry, Poland) at 4 °C for 15 days and sampled on days 2, 6, 8, 14 and 15. Before packaging (day 0) and during storage, the colour, pH, free water content and total viable counts were assessed. Additionally, at 0 days, the nutritional value of the meat was determined. Starting from the 2nd day of storage, purge loss (%) of stored raw meat was determined, and then samples were subjected to cooking, after which cooking loss and sensory quality were assessed.

### 2.2. Colour Measurement 

Colour measurement was conducted at the beginning of the experiment (day 0, before packaging of samples) and during storage (on the 2nd, 6th, 8th, 14th and 15th day). The measurement was performed using Konica Chromameter CR-400 (Sensing Inc., Osaka, Japan), D65 illuminant, 10° observer and aperture 2.54 cm. Prior to analyses, the device was calibrated using white tile. The values of lightness (L*), redness (a*) and yellowness (b*) were measured in three different locations on the surface of each pork slice. Hue (*H*) and chroma (C) were calculated [20] using Equations (1) and (2):

(1) $$H=\tan^{-1}\frac{b^{*}}{a^{*}}$$

(2) $$C=\sqrt{\left(a^{*}{}^{2}+b^{*}{}^{2}\right)}$$

The dynamic of colour changes (ΔE) during storage for each sample was calculated according to Equation (3) [21]:

(3) $$\Delta E=\sqrt{{\left(\Delta L^{*}\right)}^{2}+{\left(\Delta a^{*}\right)}^{2}+{\left({\Delta b}^{*}\right)}^{2}}$$

where ΔE is colour change; ΔL*, Δa* and Δb* are changes in L*, a* and b*, respectively. 

In the study, three types of ΔE were calculated: ΔE_0_ to describe a colour difference between fresh meat (0 days) and the next measurement on stored meat, ΔE_0,5_ to describe a colour difference between the control meat sample and the corresponding sample with the addition of 0.5% oil, and ΔE_1,0_ to describe a colour difference between the control meat sample and the corresponding sample with the addition of 1.0% oil. All ΔE were calculated using mean values of L*, a* and b*. 

The differences in Δ*E* were subdivided into six levels based on the colour difference classification by the National Bureau of Standards (Equation (4)):

(4) NBS=ΔE·0.92

and on this basis, the differences in Δ*E* among the samples were classified as 0–0.5 (trace), 0.5–1.5 (slight), 1.5–3.0 (noticeable), 3.0–6.0 (appreciable), 6.0–12.0 (much) and 12.0 or more (very much) [22].

The redness index (RI) was calculated according to AMSA [20] using Equation (5):

(5) $$RI=\frac{a^{*}}{b^{*}}$$

### 2.3. Nutritional Value

The nutritional value of pork loin was determined using an automatic analyser FoodScan Lab Meat Analyser (Foss, Hilleroed, Denmark). Pork samples (n = 9, from day 0) were minced through 6 mm mesh and placed on a glass dish in a way providing an even thickness of the meat and the absence of air holes. The proximate composition (moisture, protein, fat and collagen) was determined. 

### 2.4. pH Determinations

Values of pH were determined by directly placing the electrode of the pH meter in the meat tissue using Testo 205 pH meter (Testo, Titisee-Neustadt, Germany). Before measurements, the device was calibrated using 7.01 and 4.01 buffers. The analyses were performed in triplicate for each sample. 

### 2.5. Free Water Content Determination 

Free water was determined according to the Grau–Hamm method [23] with modifications [24]. From each pork loin sample, 50 g was cut after colour and pH determination and minced twice through 3 mm mesh. Samples of 0.3 g were prepared and placed on a glass tile on Whatman 1 filter paper (Whatman Laboratory Division, Maidstone, UK), covered with a glass tile. The weight of 2 kg was placed on the top and kept for 5 min to remove free water from the sample. Then, the filter paper with a pressed meat sample and water stain was photographed using a digital camera (Nikon D90, Nikon Corporation, Tokyo, Japan). The analysis was performed in triplicate for each meat sample. The images were analysed in image analysis software (Nikon NIS-Elements BR 2.20., Nikon Corporation, Tokyo, Japan), and free water content was calculated using Equation (6):

(6) $$\mathrm{Free}\;\mathrm{water}=\frac{\mathrm{FPabs}\cdot \left(l-m\right)}{c}\;\left[\%\right]$$

where FPabs is the absorptiveness of the filter paper (cm^3^); l is the area of liquid stain (cm^2^); m is the area of meat sample (cm^2^); c is the meat sample weight (g).

### 2.6. Purge Loss and Cooking Loss

Starting from the 2nd day of storage just after opening the packages, samples of pork were weighed to determine purge loss based on their weight before packaging and after storage. Before and after a thermal treatment (cooking in a water bath at a temperature of 80 °C to achieve 72 °C in the centre) they were weighed as well, and cooking loss was determined. Purge and cooking losses were calculated according to Equation (7):

(7) $$\mathrm{loss}=\frac{m_{1}-m_{2}}{m_{1}}\cdot 100\;\left[\%\right]$$

where m_1_ is the weight of the sample before processing (g); m_2_ the weight of the sample after treatment (g)

### 2.7. Sensory Evaluation

Before conducting the sensory assessment, the meat samples were subjected to cooking in a water bath at a temperature of 80 °C to achieve 72 °C in the centre. The temperature was monitored using a thermometer HI 93542 with an HI-766 probe (Hanna Instruments, Olsztyn, Poland). After termination of heating, samples were cut into cubes 2 cm × 2 cm and presented on white plates to panellists (n = 6). Before analyses, panellists were trained in the evaluation of pork. The following attributes were evaluated on a scale from 1 to 10 points: meat aroma intensity (1—not perceptible; 10—the strong, natural aroma of fresh meat), EO aroma intensity (1—not perceptible; 10—strong, herbal aroma), aroma (1—odorous, unacceptable; 10—pleasant smell, highly acceptable), juiciness (1—extremely dry; 10—very juicy), meat taste intensity (1—not perceptible; 10—the strong, natural taste of fresh meat), EO taste intensity (1—not perceptible; 10—strong, herbal taste) and taste (1—foreign, unacceptable; 10—pleasant taste, highly acceptable). During evaluation, water and bread were provided to cleanse the palate. During each session, a maximum of 6 samples were evaluated.

### 2.8. Microbiological Analysis

At each sampling time, just after opening the MAP packages, 10 g of sample were cut aseptically to produce decimal dilutions for microbiological analysis using 90 mL of sterile physiological liquid (0.85% of saline), followed by serial decimal solutions using 1 mL of the suspension and 9 mL of the physiological liquid. Total viable counts (TVC) were determined using Petrifilm 3M Aerobic Count Plates (Noack Group, Vienna, Austria). The samples were incubated at 35 °C ± 1 °C for 48 h [25]. After incubation, all red dots on Petrifilm plates regardless of size or intensity were counted as colonies. Numbers of TVC were reported as log_10_ colony forming units (CFU)/g.

### 2.9. Statistical Analysis

To assess the effect of the oregano essential oil addition (3 levels—0%, 0.5% and 1.0%) and time (6 levels: days 0, 2, 6, 8, 14, 15) two-way ANOVA was used. If a significant effect of oil or time was noted, then means were compared according to the following procedure. Normal distribution of data was tested using Shapiro–Wilk’s test and variance homogeneity using Levene’s test. The data showed normal distribution and variance homogeneity, and therefore variance analysis was conducted as well as Tukey’s RIR post hoc test. Sensory analysis results were analysed taking into account the effect of fixed factors (n = 2, essential oil: control, EO 0.5 and EO 1.0, and storage day: 2, 6, 8, 14, 15) and a random factor (panellist). Mean values were compared using a non-parametric Kruskal–Wallis test. All the calculations were conducted in Statistica 13.3 software (Tibco Software Inc., Palo Alto, CA, USA).

## 3. Results

### 3.1. Nutritional Value

The nutritional value of pork loins used in the study was determined to characterize a raw material. The meat contained 74.0% (±0.3%) of moisture, 22.9% (±0.2%) protein, 2.8% (±0.3%) fat and 0.8% (±0.1%) of collagen. According to the information provided by the meat processing plant, which orders monitoring of nutritional value in an accredited laboratory (Eurofins Polska Sp. z.o.o., Malbork, Poland), the meat contains below 0.5% carbohydrates, below 0.2% fibre, salt < 0.25% and sodium less than 0.1 g in 100 g.

### 3.2. The Effect of EO and Storage Time on the MAP-Packed Pork Colour

The influence of EO addition and time on pork colour is shown in Table 1, whereas changes in pork loin colour parameters in samples during storage are in Table 2. The addition of EO affected L* (*p* < 0.001), a* (*p* < 0.01) and H (*p* < 0.05) values (Table 1). The application of oregano EO in the concentration of 1.0% increased lightness and hue and decreased redness, and significant differences between control and EO 1.0 samples were noted. Oregano EO used in the concentration of 0.5% did not significantly affect pork colour. 

Storage time affected significantly all colour attributes (*p* < 0.001, Table 1). The first significant change in the pork colour (L*, a*, b*, C) was noted after 2 days of storage. Lightness increased continuously, reaching the highest values on day 15. Redness increased between the 0 and 2nd day and then started to decrease slowly. Between the 6th and 14th day, there were no significant differences in a* values. The lowest a* values were noted on day 15 of storage. Yellowness (b*) increased significantly (*p* < 0.05) between the 0 and 2nd day, whereas between the 6th and 15th day, it remained unchanged. A similar trend was noted for C values, whereas hue (H) increased over time. As a result of changes in a* and b* values, significant changes in RI appeared, and it started to decrease from day 2; however, there were no significant changes between the 8th, 14th and 15th day of storage.

A significant interaction between EO and time was noted for L* and a* values (*p* < 0.05). The addition of 1% EO caused a significant reduction in a* values, below the value noted for fresh pork at the beginning of the experiment. In control samples, a significant increase in L* between the 14th and 15th day was noted, in contrast to oregano EO-containing samples in which the values did not differ (Table 2). 

### 3.3. Colour Changes between Fresh (0 Days) and Stored Pork

Changes in the pork colour during storage were interpreted based on the ΔE coefficient. It was noted the changes were appreciable in all samples after two days of storage. In the next sampling days (6th, 8th and 14th), changes in colour were much, whereas on day 15 in control and EO 1.0 very much and in EO 0.5 much. The interpretation of the ΔE coefficient enables us to conclude that as little time as 2 days of storage changes meat’s colour to the extent that the colour will be identified as different from fresh meat colour (Table 3). Between the 14th and 15th days, all colour parameters and the dynamics of colour changes indicate significant changes.

### 3.4. Colour Changes between Control and EO Samples in Each Sampling Time 

The ΔE coefficient indicated that the change in the colour between control and EO 0.5 samples on day 2 was appreciable, whereas from day 8 to 15 it was much. The application of 1% of EO produced significant differences in the colour between the control and EO 1.0 samples—differences noted after 2, 6, 8 and 14 days of storage were classified as much, whereas after 15 days of storage as very much. Based on ΔE it might be concluded that the addition of oregano EO did not produce such a colour difference that would be identified by consumers who would evaluate control and EO-containing pork samples, except for EO 1.0 samples after 14 d storage, where ΔE exceeded 3 (3.41, Table 3).

### 3.5. MAP Composition, pH, Free Water, Purge and Cooking Losses

During storage, the MAP composition did not change. Values of pH determined on samples with different oregano EO concentrations at different sampling times ranged from 5.3 to 5.7. The application of the EO onto the surface of pork samples did not result in changes in pH values, free water content, purge loss or cooking loss (Table 4). In contrast, values of pH, free water and purge loss were affected by storage time. A significant (*p* < 0.05) decrease in pH was noted between 0 and the 2nd day, then pH remained stable until the 8th day, when it started to decrease again, which produced differences in pH determined on the 8th and 15th day. Free water content decreased (*p* < 0.05) between 0 and the 6th day, and no further changes were noted up to the 15th day (Table 4). An increase in purge loss between the 2nd and the 6th day and between the 8th and the 15th day of storage was noted (*p* < 0.05), which corresponded with the decrease in free water content.

### 3.6. Sensory Quality

The application of essential oil affected meat aroma and taste intensity and herbal aroma and taste intensity (Table 5), whereas other attributes of sensory quality remained unaffected. As expected with the increased proportion of EO used, the meat aroma intensity decreased (*p* < 0.001), whereas herbal aroma (*p* < 0.05) and herbal taste (*p* < 0.001) increased. However, in terms of meat taste intensity, EO 0.5 samples were scored similarly to the control, whereas EO 1.0 samples gained lower scores. 

Storage time affected the quality of pork in terms of aroma and herbal aroma intensity. Due to the fact that on days 14 and 15, aroma scored low, which indicated that the meat was not fresh and suitable for consumption, the remaining attributes of the sensory quality were not evaluated. Herbal aroma intensity decreased, and significant differences were noted between days 6 and 15; however, no differences occurred up to the 14th day. The effect of storage time (from the 2nd to the 8th day) was noted in taste, which decreased from the 6th and 8th day.

### 3.7. Total Viable Counts

**Figure 1:**
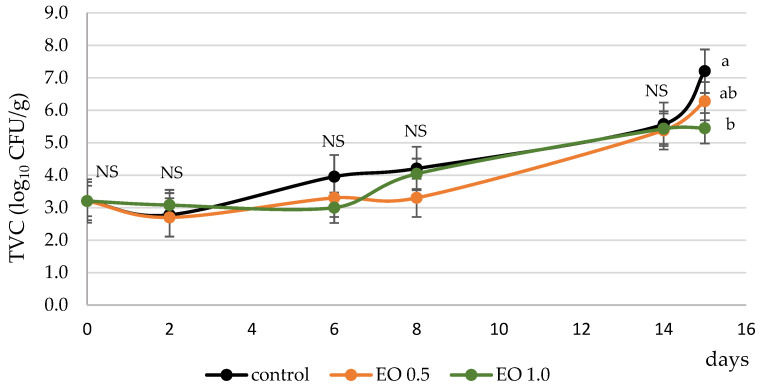
Total viable counts (TVC, log_10_ CFU/g) in MAP-packed pork without oregano essential oil addition (control) and with 0.5% (EO 0.5) and 1% (EO 1.0) of oregano essential oil. a-b—values with different letters differ significantly at *p* < 0.05, NS—non-significant differences between treatments at the same sampling point.

At the beginning of the experiment, the TVC was at the level of 3 log_10_ CFU/g and increased during the experiment (Figure 1). The TVC remained below 7 log_10_ CFU/g in all treatments until day 14; however, the next day it increased and reached 7 log_10_ CFU/g in control samples. On day 15, TVC in control differed significantly from EO 1.0, in which it was 5.4 log_10_ CFU/g (Figure 1). Up to the 14th day, there were no differences between treatments (*p* > 0.05), which indicated that oregano EO used in the concentration of 0.5% and 1.0% failed to inhibit bacterial growth in MAP-packed pork.

## 4. Discussion

### 4.1. Colour

Meat colour is one of the most important quality attributes because the appearance of raw meat, especially its colour, is the first thing that is evaluated by a consumer when making a decision about a purchase [26,27,28,29]. Even small differences in the colour are related to the probability of a purchase [30]. The colour of fresh meat depends on the concentration and physical status of myoglobin (deoxymyoglobin gives a dark red colour, oxymyoglobin bright red and metmyoglobin brown colour). Generally, the intensive (dark or light) red colour (resulting from a high proportion of oxymyoglobin) of pork is regarded as desirable, because it is associated with freshness and the highest quality of the meat [21,26,27,30,31]. During meat storage, oxymyoglobin and deoxymyoglobin undergo oxidation, which results in an increase in metmyoglobin proportion, which makes the meat colour brown and less attractive to consumers [32,33]. In the present study, it was noted that changes in L* and a* colour attributes were affected by EO concentration and storage time, with a significant interaction between these factors, whereas b*, C, H and RI were affected by storage time only. This is related to protein denaturation, which induces an increase in the L* parameter in meat [30], while oxidation and metmyoglobin formation cause a decrease in a* [34] and consequently an increase in b* values [35].These findings are similar to those reported by Rosa et al. [36] who studied changes in pork loin quality during storage in MAP and found out that L* and a* were affected by storage time (*p* < 0.01) and also by MAP composition. 

In the present study, the first significant change (*p* < 0.05) in L*, a*, b* and C attributes was noted after 2 days of storage. An increase in a* corresponded to an increase in meat redness. It resulted from the higher availability of oxygen in MAP packaging (70% O_2_/25% CO_2_/5% N_2_), which produced the switch of myoglobin into oxymyoglobin [32]. A continuous increase in the lightness was noted as well in the present study, which might be caused by differences in the proportion of reflected absorbed light. According to Li et al. [37], this phenomenon results from protein denaturation. As a result of changes in the protein structure, moisture migrates to the surface of meat increasing the coefficient of light reflection. The trend towards the increase in L* values during the whole period of meat storage was noted also by Chouliara et al. [16], who applied oregano EO in the concentration of 1% on the surface of chicken breast muscles. The reduction in redness (a*) and increase in lightness (L*) in pork meat during storage results also from all the changes related to the process of meat spoilage, including protein denaturation, lipid oxidation and pH modification [33]. In the present study, a significant decrease (*p* < 0.05) in a* in samples treated with 1% oregano EO was noted on day 15. It might be caused by an interaction of EO with myoglobin and a decrease in the attractiveness of raw meat. Similar results were reported by Paparella et al. [17], who used 2% oregano EO on pork stored for 15 days. 

The newest reports [28,29] indicate that changes in colour might be described based on redness (a*) and yellowness (b*) values, which change the most dynamically during storage and are closely correlated with myoglobin changes. Other useful attributes are chroma (C) and hue (H), which are calculated using a* and b* values and are well-correlated with colour perception by the human eye. However, to study the colour differences in time, the ΔE* coefficient might be successfully applied, instead of studying the evolution of each colour attribute separately. ΔE* enables the description of a colour change between two colour stimuli, which is best associated with the human ability to distinguish differences in colour [28,29]. Altmann et al. [28] investigated the ability of consumers to differentiate the continuously changing colour of fresh pork using a computer vision system. For this purpose, a centralised, computer-assisted, systematic discrimination method was used. It was proved that the total colour difference (ΔE) of 1 is a threshold for colour difference recognition for an observer. Values of ΔE between 1 and 2 are suggested as essential to notice differences in colour during product sorting and to monitor their colour as necessary criteria for meat product quality for the experienced observer (meat processing plant staff) [28]. Although it is known that ΔE > 2 indicates colour changes visible also for inexperienced observers, it is assumed that changes in colour will be noticeable for consumers if ΔE > 3 [29]. Ultimately, when ΔE > 5, an observer has a sensation of two different colours [27,38]. To associate a colour difference recorded by a spectrophotometer with a food product, all data were re-calculated to NBS units, the interpretation of which was presented in Section 2 [38].

The efficient application of ΔE to estimate the time needed for the stabilization of colour attributes during beef ageing was presented in the paper by Tkacz et al. [27]. Because the colour stability during storage or exposition in a store is highly desirable by consumers and producers [30], to analyse the results of the present study, ΔE was used as a colour difference between fresh pork (packed at day 0) and pork stored for 2,4,6,8,14 and 15 days. When ΔE results have described the value of ΔE = 3, it was used as a threshold, and if ΔE values were below 3, they were described as imperceptible, whereas those above 3 were significant. It was noted that storage of pork under conditions used in the present study affects the meat’s colour—the change is noticeable just after 2 days of storage. The application of oregano EO in the concentration of 0.5% and 1.0% did not change it. The results indicate also that during the remaining days of storage, a consumer would not be able to distinguish the colour of control samples from those containing EO, except for EO 1.0 samples on the 14th day.

### 4.2. pH and Expressible Water

The values of pH noted at the beginning of the experiment (day 0) were typical for normal-quality meat without PSE or DFD defects [39]. Values of pH decreased during storage, which was noted also by Boskovic et al. [13]. In MAP-packed meat during storage, CO_2_ dissolves in water, and a weak carbonic acid is produced, which explains the pH decrease [15]. 

The ability of meat to retain water during transportation, storage and processing is described as water-holding capacity (WHC) [23]. It has an impact on the economic value and quality of meat because it affects not only fresh meat weight changes at every stage of distribution and processing but also its sensory quality, including juiciness and tenderness [40]. The water-holding capacity of meat might be assessed based on different analyses such as free water content, drip or purge loss, and cooking loss. Taking into consideration changes in free water content, it was noted that it decreased in time, which indicates that the ability of muscle tissue increased. This is a well-described phenomenon, which results from changes in the myofibrillar, including cytoskeletal proteins and structure [41]. However, this lower content of free water might also result from the purge loss that occurred during storage. In fact, purge loss increased in time, which might be explained by a decrease in pH. When the pH value of meat shifts towards the isoelectric point of muscle proteins, the water-holding capacity of meat decreases [39]. Due to the fact that EO addition did not affect pH, the water-holding capacity of meat was unchanged. 

### 4.3. Sensory and Microbiological Quality

Results of the sensory analysis indicated that the application of oregano essential oil in the proportion of 0.5% did not affect meat taste intensity, although the aroma and taste of oregano EO were noticeable for consumers. Due to the lack of differences between control and EO 0.5 and EO 1.0 samples in terms of quality of aroma and taste, it might be concluded that the application of oregano essential oils does not have a negative effect on these attributes and might be applied on the meat surface to obtain new attractive products with a well-detectable herbal aroma and taste. On the other hand, the oregano essential oil used in the concentrations of 0.5% and 1.0% did not cover the unpleasant stale aroma of meat, which indicates that consumers still will be able to judge if the meat is suitable for consumption. The deterioration of meat aroma occurred between the 8th and 14th day and was associated with the increase in TVC. As a result of bacteria metabolism, objectionable compounds are created, including those causing off-odours, gas and slime, which decrease the quality of meat products in terms of taste, aroma and colour [19]. 

The oregano essential oil used on the surface of pork did not show an inhibitory effect against TVC up to the 14th day of storage; however, the effect was noted on the 15th day. Taking into consideration that the shelf-life of pork packed in MAP is 14 days (as set by a producer), the inhibitory effect noted on day 15 is too weak to preserve the pork quality. Based on the TVC increase in MAP-packed pork during storage, it might be concluded that oregano EO used in the concentration of 0.5% and 1.0% on the surface of the meat did not exhibit an antimicrobial activity enough to preserve the meat. The increase in TVC during pork storage was noted also in the study by Hao et al. [19], who studied the effect of an active coating with oregano essential oil on pork quality. In their study, TVC increased sharply in control samples, whereas in samples packed in the active coating, the growth was inhibited. On the other hand, the number of 6 log_10_ CFU/g (which was indicated by Hao et al. [19] as a threshold value for fresh meat) was noted between the 9th and 12th day, whereas in the present study, TVC was below the threshold up to the 14th day. The results highlight the importance of good hygienic conditions during slaughter and post-slaughter carcass processing (the initial TVC in the present study was 1 log cycle lower than in the study of Hao) as well as the effectiveness of MAP in bacterial growth inhibition. Differences in the antimicrobial effectiveness of EO were also noted by van Haute et al. [6], who concluded that the packaging method should always be incorporated in the experimental design as a significant factor. The antimicrobial effectiveness of EO is affected also by pH and the presence of other antioxidants. It was shown that the reduced pH increased the hydrophobicity of essential oils, which resulted in their attachment to the pathogen’s lipid cell membrane [42]. EO might affect bacterial metabolism by, e.g., binding on membrane proteins and inhibiting certain necessary enzymes [43]. Essential oils differ in their effectiveness even when used under the same conditions. In the study of Mantzourani et al. [44], oregano EO was used in combination with wine to marinate the pork. In comparison with other treatments, such as wine, wine mix and wine with thyme EO, the oregano EO-containing treatment was the most effective in inhibiting bacterial growth. However, no significant differences between the treatment with oregano EO and thyme EO were noted in the shelf-life of marinated pork. 

Oregano essential oil is known for its high efficiency in bacterial growth inhibition. In the study of Man et al. [45], its inhibitory effect on the most common pathogenic bacteria (*Staphylococcus aureus*, *Enterococcus faecalis*, *Escherichia coli*, *Klebsiella pneumonia*, *Pseudomonas aeruginosa*) was reported when used in the form of micelle suspensions in an in vitro study. In the present study, the oregano essential oil effectiveness was tested in the raw meat environment stored in the MAP, and a slight inhibitory effect of the oregano oil was noted. The lack of the strong antimicrobial effect of the oregano essential oil on TVC noted in the present study might result from a low concentration obtained in the pork surface and MAP composition used in the study. Skandamis and Nychas [46] noted that the inhibitory potential of essential oregano oil depended on the method the meat was packed and stored. It was the lowest when meat samples were stored in the air and increased in the following order: vacuum < 100% CO_2_ < MAP 40% CO_2_, 30% N_2_, 30% O_2_. In the present study, MAP contained as much as 70% oxygen. As pointed out by Zhang and Piao [47], there are some factors that limit the application of essential oils in meat and meat products, including their sensitivity to light and oxygen. Other limiting factors are interactions of essential oils with lipophilic components of the meat matrix (fats and protein), high volatility and low solubility in the aqueous phase [47,48]. Therefore, it might be concluded that in the atmosphere with such a high oxygen proportion as used in the study, the oregano EO partially lost its activity, which resulted in a slight antimicrobial effect and failure to prevent meat surface discolouration during storage.

## 5. Conclusions

The application of oregano EO in the concentration of 0.5% and 1.0% did not prevent unfavourable colour changes in the MAP pork; therefore, it cannot be used as a method for colour preservation. The concentrations chosen based on their effect on the sensory quality of meat (0.5% and 1.0%) turned out to be too low to exert colour-protective and antimicrobial effects. On the other hand, they did not cover the symptoms of meat spoilage, which might be dangerous for consumers. Nevertheless, oregano essential oil might be used in fresh pork to obtain a new product with a specific herbal aroma and taste, with modifications in the water-holding capacity of the meat. Further studies are needed to develop new methods to limit changes in fresh meat colour packed in MAP, which is highly desirable by the meat industry. 

## Figures and Tables

**Table 1:** The effect of oregano essential oil (EO) and storage time on the colour of pork loin (mean value ± standard error of the mean).

Attribute	Essential Oil (EO)			Time (T, days)						*p*-Value		
	C	EO 0.5	EO 1.0	0	2	6	8	14	15	EO	T	EOxT
L*	47.77 ± 0.65 ^y^	48.77 ± 0.46 ^y^	49.70 ± 0.64 ^x^	44.76 ± 0.21 ^e^	48.91 ± 0.69 ^d^	51.78 ± 0.46 ^c^	52.94 ± 0.53 ^bc^	54.45 ± 0.54 ^b^	56.81 ± 0.43 ^a^	***	***	**
a*	1.70 ± 0.07 ^x^	1.61 ± 0.08 ^xy^	1.46 ± 0.06 ^y^	1.57 ± 0.04 ^b^	2.08 ± 0.13 ^a^	1.71 ± 0.09 ^ab^	1.76 ± 0.11 ^ab^	1.36 ± 0.08 ^bc^	1.22 ± 0.08 ^c^	**	***	**
b*	6.66 ± 0.25 ^x^	7.01 ± 0.27 ^x^	7.23 ± 0.28 ^x^	4.98 ± 0.10 ^c^	7.52 ± 0.19 ^b^	8.40 ± 0.20 ^ab^	9.62 ± 0.27 ^ab^	9.84 ± 0.16 ^ab^	10.12 ± 0.09 ^a^	NS	***	NS
C	6.90 ± 0.29 ^x^	7.23 ± 0.26 ^x^	7.41 ± 0.28 ^x^	5.24 ± 0.10 ^c^	7.82 ± 0.19 ^b^	8.58 ± 0.20 ^ab^	9.79 ± 0.28 ^ab^	9.94 ± 0.16 ^ab^	10.20 ± 0.08 ^a^	NS	***	NS
H	74.42 ± 0.75 ^y^	75.71 ± 0.70 ^xy^	77.19 ± 0.67 ^x^	72.23 ± 0.49 ^c^	74.49 ± 0.92 ^c^	78.53 ± 0.51 ^b^	79.73 ± 0.54 ^ab^	82.11 ± 0.48 ^ab^	83.09 ± 0.44 ^a^	*	***	NS
RI	0.28 ± 0.01 ^x^	0.26 ± 0.01 ^x^	0.23 ± 0.01 ^x^	0.32 ± 0.01 ^a^	0.28 ± 0.02 ^a^	0.20 ± 0.01 ^b^	0.18 ± 0.01 ^bc^	0.14 ± 0.01 ^bc^	0.12 ± 0.01 ^c^	NS	***	NS

^x,y^—mean values in rows within EO with a common letter do not differ significantly at *p* < 0.05. ^a–e^—values in rows with different letters differ significantly at *p* < 0.05. *** a difference significant at *p* < 0.001. ** a difference significant at *p* < 0.01. * a difference significant at *p* < 0.05. NS—non-significant difference *p* > 0.05. C—control, without EO. EO 0.5—samples with the addition of EO in a concentration of 0.5%. EO 1.0—samples with the addition of EO in a concentration of 1.0%.

**Table 2:** Changes in pork loin colour parameters during storage (mean value ± standard error of the mean).

Colour Parameters	0 Days	2 Days	6 Days	8 Days	14 Days	15 Days
The colour of meat without the addition of essential oil (C)						
L*	44.37 ± 0.34 ^d^	48.11 ± 0.80 ^c^	51.33 ± 0.66 ^bc^	52.59 ± 0.17 ^b^	52.05 ± 1.39 ^b^	57.39 ± 0.91 ^a^
a*	1.60 ± 0.06 ^b^	2.52 ± 0.21 ^a^	1.62 ± 0.14 ^ab^	2.30 ± 0.14 ^ab^	1.31 ± 0.08 ^b^	1.59 ± 0.08 ^ab^
b*	5.00 ± 0.16 ^b^	7.37 ± 0.24 ^ab^	8.29 ± 0.21 ^a^	10.32 ± 0.05 ^a^	9.33 ± 0.36 ^a^	9.96 ± 0.06 ^a^
C	5.27 ± 0.15 ^b^	7.80 ± 0.25 ^a^	8.45 ± 0.22 ^a^	10.57 ± 0.07 ^a^	9.42 ± 0.36 ^a^	10.09 ± 0.08 ^a^
H	71.72 ± 0.85 ^b^	71.15 ± 1.48 ^b^	78.95 ± 0.87 ^a^	77.45 ± 0.70 ^ab^	82.00 ± 0.52 ^a^	80.95 ± 0.42 ^a^
RI	0.33 ± 0.02 ^a^	0.34 ± 0.03 ^a^	0.20 ± 0.02 ^b^	0.22 ± 0.01 ^ab^	0.14 ± 0.01 ^c^	0.16 ± 0.01 ^bc^
Meat colour with the addition of 0.5% essential oil (EO 0.5%)						
L*	45.18 ± 0.44 ^b^	47.64 ± 1.34 ^ab^	51.67 ± 0.76 ^a^	52.19 ± 0.87 ^a^	54.56 ± 0.61 ^a^	55.04 ± 0.58 ^a^
a*	1.63 ± 0.09 ^ab^	2.34 ± 0.25 ^a^	1.58 ± 0.20 ^ab^	1.60 ± 0.15 ^ab^	1.49 ± 0.10 ^ab^	1.29 ± 0.11 ^b^
b*	5.07 ± 0.19 ^b^	7.40 ± 0.36 ^ab^	8.27 ± 0.21 ^a^	9.15 ± 0.46 ^a^	9.98 ± 0.26 ^a^	9.93 ± 0.11 ^a^
C	5.35 ± 0.20 ^b^	7.76 ± 0.41 ^ab^	8.43 ± 0.21 ^a^	9.29 ± 0.47 ^a^	10.10 ± 0.27 ^a^	10.01 ± 0.11 ^a^
H	71.89 ± 0.85 ^c^	72.63 ± 1.18 ^bc^	79.23 ± 1.30 ^ab^	80.19 ± 0.72 ^a^	81.54 ± 0.53 ^a^	82.63 ± 0.60 ^a^
RI	0.33 ± 0.02 ^a^	0.31 ± 0.02 ^ab^	0.19 ± 0.02 ^bc^	0.17 ± 0.01 ^c^	0.15 ± 0.01 ^c^	0.13 ± 0.01 ^c^
Meat colour with the addition of 1.0% essential oil (EO 1.0%)						
L*	44.63 ± 0.26 ^e^	50.05 ± 1.12 ^d^	52.16 ± 0.89 ^cd^	54.50 ± 0.42 ^bc^	55.40 ± 0.87 ^ab^	58.19 ± 0.22 ^a^
a*	1.46 ± 0.08 ^ab^	1.70 ± 0.13 ^ab^	1.86 ± 0.15 ^a^	1.72 ± 0.16 ^ab^	1.24 ± 0.15 ^bc^	0.91 ± 0.07 ^c^
b*	4.85 ± 0.13 ^c^	7.68 ± 0.33 ^b^	8.56 ± 0.43 ^b^	10.06 ± 0.13 ^a^	9.93 ± 0.23 ^a^	10.43 ± 0.17 ^a^
C	5.09 ± 0.13 ^c^	7.88 ± 0.33 ^b^	8.76 ± 0.45 ^b^	10.21 ± 0.10 ^a^	10.02 ± 0.21 ^a^	10.47 ± 0.16 ^a^
H	73.11 ± 0.86 ^c^	77.39 ± 0.99 ^bc^	77.78 ± 0.63 ^b^	80.26 ± 0.98 ^ab^	82.73 ± 1.02 ^ab^	84.98 ± 0.44 ^a^
RI	0.31 ± 0.02 ^a^	0.22 ± 0.02 ^a^	0.22 ± 0.01 ^a^	0.17 ± 0.02 ^a^	0.13 ± 0.02 ^b^	0.09 ± 0.01 ^c^

^a–e^—values in rows with different letters differ significantly at *p* < 0.05. L*—lightness, a*—redness, b*—yellowness, C—chroma*, H—hue angle*, RI—redness index [a*/b*].

**Table 3:** Dynamics of meat colour changes during storage pork loin (mean value ± standard error of the mean).

Colour Change Dynamics	0 Days	2 Days	6 Days	8 Days	14 Days	15 Days
The colour of meat without the addition of essential oil (C)						
ΔE_0_	-	4.52	7.70	9.81	8.82	13.93
NBS_0_	-	4.14	7.08	9.03	8.12	12.81
Meat colour with the addition of 0.5% essential oil (EO 0.5%)						
ΔE_0_	-	3.47	7.24	8.11	10.60	11.00
NBS_0_	-	3.19	6.66	7.46	9.75	10.12
Meat colour with the addition of 1.0% essential oil (EO 1.0%)						
ΔE_0_	-	6.12	8.40	11.16	11.91	14.67
NBS_0_	-	5.63	7.73	10.27	10.96	13.50
ΔE_0.5_	-	0.50	0.35	1.42	2.60	2.36
NBS_0.5_	-	0.46	0.32	1.31	2.39	2.18
ΔE_1.0_	-	2.12	0.91	2.01	3.41	1.16
NBS_1.0_	-	1.95	0.84	1.85	3.13	1.06

ΔE_0_—the colour difference between fresh meat (0 days) and the next measurement on stored meat. ΔE_0.5_—the colour difference between the control meat sample and the corresponding sample with the addition of 0.5% oil. ΔE_1.0_—the colour difference between the control meat sample and the corresponding sample with the addition of 1.0% oil. NBS—National Bureau of Standards.

**Table 4:** The effect of oregano essential oil (EO) and storage time on pH and free water in pork loin (mean value ± standard error of the mean).

Attribute	Essential Oil (EO)			Time (T, Days)						*p*-Value		
	C	EO 0.5	EO 1.0	0	2	6	8	14	15	EO	T	EOxT
pH	5.48 ± 0.01 ^x^	5.47 ± 0.01 ^x^	5.48 ± 0.01 ^x^	5.56 ± 0.00 ^a^	5.40 ± 0.01 ^b^	5.41 ± 0.01 ^b^	5.40 ± 0.01 ^b^	5.39 ± 0.01 ^bc^	5.35 ± 0.01 ^c^	NS	***	NS
Free water (%)	25.94 ± 0.59 ^x^	25.49 ± 0.50 ^x^	24.67 ± 0.45 ^x^	28.07 ± 0.93 ^a^	26.88 ± 0.71 ^ab^	24.31 ± 0.60 ^bc^	25.53 ± 0.93 ^abc^	23.55 ± 0.34 ^c^	24.60 ± 0.52 ^bc^	NS	***	NS
Purge loss (%)	9.38 ± 0.92 ^x^	9.27 ± 0.98 ^x^	8.60 ± 0.77 ^x^	ND	5.54 ± 0.55 ^c^	9.08 ± 0.66 ^b^	9.78 ± 0.70 ^b^	11.58 ± 0.53 ^ab^	13.42 ± 0.25 ^a^	NS	***	NS
Cooking loss (%)	22.63 ± 1.13 ^x^	23.78 ± 1.17 ^x^	23.73 ± 1.08 ^x^	ND	24.04 ± 1.54 ^a^	23.63 ± 0.89 ^a^	21.68 ± 1.24 ^a^	22.40 ± 1.60 ^a^	26.26 ± 0.30 ^a^	NS	NS	NS

^x^—mean values in rows within EO with a common letter do not differ significantly at *p* < 0.05; ^a–c^—mean values in rows within storage days with different letters differ significantly at *p* < 0.05; *** a difference significant at *p* < 0.001; NS—non-significant difference *p* > 0.05; C—control, without EO; EO 0.5—samples with the addition of EO in a concentration of 0.5%; EO 1.0—samples with the addition of EO in a concentration of 1.0%; ND—not determined.

**Table 5:** Sensory quality of pork packed in the modified atmosphere with 0.5% and 1% of oregano essential oil (EO) and without the essential oil (C) (mean values ± standard error of the mean).

Attribute	Essential Oil (EO)				Time (T, D)				*p*-Value		
	C	EO 0.5	EO 1.0	2	6	8	14	15	EO	S	
Aroma (quality)	4.8 ± 0.3	5.2 ± 0.3	5.4 ± 0.3	6.4 ^a^ ± 0.3	6.1 ^a^ ± 0.3	5.5 ^a^ ± 0.3	2.4 ^b^ ± 0.4	2.0 ^b^ ± 0.4	NS	**	NS
Meat aroma intensity	6.7 ^x^ ± 0.2	4.8 ^y^ ± 0.3	3.5 ^z^ ± 0.2	5.2 ± 0.4	5.3 ± 0.3	5.2 ± 0.4	5.4 ± 0.5	3.7 ± 0.5	***	NS	NS
Herbal aroma intensity	1.6 ^z^ ± 0.3	5.2 ^y^ ± 0.3	6.9 ^x^ ± 0.2	4.6 ^a^ ± 0.5	4.7 ^a^ ± 0.5	3.8 ^ab^ ± 0.4	3.0 ^ab^ ± 0.4	2.0 ^b^ ± 0.4	*	*	NS
Juiciness	5.6 ± 0.2	5.3 ± 0.3	5.1 ± 0.3	5.8 ± 0.2	5.7 ± 0.3	4.5 ± 0.3	ND	ND	NS	NS	NS
Tenderness	5.0 ± 0.3	5.3 ± 0.4	5.2 ± 0.4	4.9 ± 0.3	5.4 ± 0.4	5.0 ± 0.4	ND	ND	NS	NS	NS
Taste (quality)	5.1 ± 0.3	5.4 ± 0.3	5.2 ± 0.2	6.0 ^a^ ± 0.3	5.5 ^a^ ± 0.3	4.0 ^b^ ± 0.3	ND	ND	NS	*	NS
Meat taste intensity	6.4 ± 0.3	5.3 ± 0.2	4.0 ± 0.2	5.4 ± 0.3	5.5 ± 0.3	5.1 ± 0.3	ND	ND	***	NS	NS
Herbal taste intensity	1.6 ^y^ ± 0.2	5.1 ^x^ ± 0.3	6.6 ^x^ ± 0.2	4.4 ± 0.5	4.6 ± 0.5	3.7 ± 0.4	ND	ND	***	NS	NS

^x,y,z^—values in rows with different letters differ significantly at *p* < 0.05. ^a,b^—values in rows with different letters differ significantly at *p* < 0.05; ND—not determined; * a difference significant at *p* < 0.05; ** a difference significant at *p* < 0.01; *** a difference significant at *p* < 0.001; NS—non-significant difference *p* > 0.05; Int.—interaction; C—control, without EO; EO 0.5—samples with the addition of EO in a concentration of 0.5%; EO 1.0—samples with the addition of EO in a concentration of 1.0%.

## Data Availability

Datasets generated from the current experiment are available from the corresponding authors upon reasonable request.
